# Resequencing microarray probe design for typing genetically diverse viruses: human rhinoviruses and enteroviruses

**DOI:** 10.1186/1471-2164-9-577

**Published:** 2008-12-01

**Authors:** Zheng Wang, Anthony P Malanoski, Baochuan Lin, Carolyn Kidd, Nina C Long, Kate M Blaney, Dzung C Thach, Clark Tibbetts, David A Stenger

**Affiliations:** 1 Center for Bio/Molecular Science & Engineering, Naval Research Laboratory, Washington, DC 20375, USA; 2 Nova Research Inc, Alexandria, VA 22308, USA; 3 Tessarae, LLC, Potomac Falls, VA 20165, USA

## Abstract

**Background:**

Febrile respiratory illness (FRI) has a high impact on public health and global economics and poses a difficult challenge for differential diagnosis. A particular issue is the detection of genetically diverse pathogens, i.e. human rhinoviruses (HRV) and enteroviruses (HEV) which are frequent causes of FRI. Resequencing Pathogen Microarray technology has demonstrated potential for differential diagnosis of several respiratory pathogens simultaneously, but a high confidence design method to select probes for genetically diverse viruses is lacking.

**Results:**

Using HRV and HEV as test cases, we assess a general design strategy for detecting and serotyping genetically diverse viruses. A minimal number of probe sequences (26 for HRV and 13 for HEV), which were potentially capable of detecting all serotypes of HRV and HEV, were determined and implemented on the Resequencing Pathogen Microarray RPM-Flu v.30/31 (*Tessarae RPM-Flu*). The specificities of designed probes were validated using 34 HRV and 28 HEV strains. All strains were successfully detected and identified at least to species level. 33 HRV strains and 16 HEV strains could be further differentiated to serotype level.

**Conclusion:**

This study provides a fundamental evaluation of simultaneous detection and differential identification of genetically diverse RNA viruses with a minimal number of prototype sequences. The results demonstrated that the newly designed RPM-Flu v.30/31 can provide comprehensive and specific analysis of HRV and HEV samples which implicates that this design strategy will be applicable for other genetically diverse viruses.

## Background

Human febrile respiratory illness (FRI) results in significant annual health and economic burden worldwide, but the diversity and number of pathogens make differential diagnosis very challenging. Thus, it represents a useful example where many organisms ranging from bacteria (*Haemophilus influenzae*) to fairly conserved viruses (respiratory syncytial virus) to genetically diverse viruses, i.e. influenza A virus, human rhinoviruses (HRV), and human enteroviruses (HEV) need to be detected for successful differential diagnosis. Several technologies, Masscode™ multiplex RT-PCR system [1], electrospray ionization mass spectrometry analysis of PCR amplicons [2], Luminex^® ^xMAP™ [3], and various microarray-based approaches [4-8], are currently under development as diagnostic platforms to effectively and simultaneously detect and identify large numbers of diverse viral and bacterial respiratory pathogens. One high-density resequencing microarray platform, the Respiratory Pathogen Microarray version 1 (RPM v.1), has been successfully demonstrated to identify a much broader range of pathogens (including bacteria and DNA and RNA viruses) in a single test at sensitivities and specificities that are similar to or improved over those of other technologies [9,10]. In addition, the RPM v.1 platform has the demonstrated capability to discriminate among known and previously unknown strains and variants of targeted pathogens [11,12].

While promising, the RPM v.1 platform was a proof-of-concept microarray for the detection of 26 common respiratory pathogens primarily encountered among military basic trainees. It did not provide comprehensive coverage of all potential respiratory pathogens and the design methodology used was not appropriate for genetically diverse viruses. The design methodology for the RPM v.1 microarray consisted of applying selection rules developed for long oligonucleotide microarrays. These rules were not optimal but worked for bacterial organisms and fairly conserved viruses since previous studies had shown a single sequence on a resequencing microarray could reliably detect and serotype strains with as much as 10 to 15% variation [8,10-12]. Their application to cover more diverse viral organisms was less successful. For example, the 5' untranslated region (5'UTR) sequence chosen for HRV on the RPM v.1 only provided identification of the prototype HRV-89 and very little coverage of other HRV serotypes. The 5'UTR sequences, which are relatively conserved among HRV and HEV, have been used in PCR and *de novo *sequencing for tentative viral identification or serotype classification in lieu of the much more variable capsid proteins that actually determine serotypes [13,14]. However, the 5'UTR sequences still have ~5 to 30% nucleotide sequence variations among different serotypes so require more than one prototype sequence for proper identification and serotyping. Serotyping HRV and HEV is important to FRI differential diagnosis because even though these "common cold" viruses generally only induce mild symptoms, they can cause a wide variety of other severe illnesses, such as aseptic meningitis [15], bronchitis and asthma [16].

New resequencing pathogen microarray designs, versions 3.0 and 3.1 (RPM-Flu v.30/31), have been constructed to address the shortcomings of the previous design. The use of 8 μm feature allows microarrays with greater coverage, currently 86, of common respiratory organisms and high human health risk zoonotic pathogens (bacteria and viruses). A new approach to select a minimal number of prototype sequences that can be used to detect all and correctly identify many of the relevant strains of genetically diverse viruses such as HRV and HEV was developed. Due to the great genetic diversity of HRV and HEV, in order to ensure that designed probes (referred to as probe sequences) generated from selected database sequences (referred to as prototype regions) would detect and discriminate all serotypes of HRV and HEV, a predictive model was used to assist the microarray design [17]. This *in silico *model developed for predicting resequencing microarray hybridization patterns shows good concordance in the overall percentage of base calls predicted versus experimental results. Thus it is possible to use this model for evaluating the performance of database sequences as potential prototype regions. In this study, we report on results of this algorithm applied to the 5'UTR sequences of HRV and HEV and confirm that using ~15% of the RPM-Flu v.30/31 microarray (17,335 HRV and HEV nucleotides of total 117,254 nucleotides on array) is sufficient to detect and differentiate many HRV and HEV serotypes.

## Results

### Microarray design process

#### *In silico *modeling

**Figure 1:**
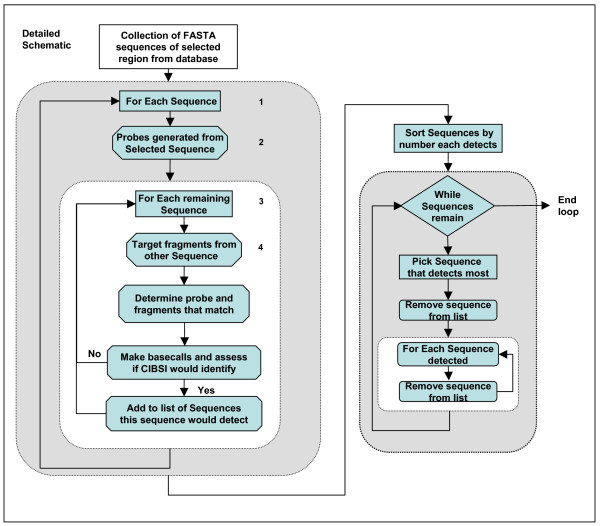
**Schematic of algorithm representing the prototype sequences selection process.** A collection of database sequences covering a specified region are processed together. Each sequence is treated as probe sequence that the other sequences are tested against. The numbers of these sequences detected by the probe sequences are determined. A group of sequences that are predicted to detect all the sequences is then selected.

Figure 1 illustrates the procedures used for the selection of HRV and HEV probe sequences. First, sequences that contain the specified target region (5'UTR) and meet any selection criteria applied were downloaded from a database (currently GenBank). Downloaded sequences were trimmed to cover the same region using pair-wise sequence alignment. These sequences were treated as target sequences (what would be detected by the microarray) and also as prototype sequences (the potential probe sequences tiled on the microarray). Each downloaded sequence was treated as a prototype used to generate probe sequences (Fig 1. Step 1–2) and the remaining sequences were treated as target sequences (Fig 1. Step 3–4). Sets of 4 25-mer probes (1 perfect match and 3 mismatches in the 13^th ^position) were generated from a prototype sequence and correspond to what would actually appear on a resequencing microarray. The other sequences were treated as a target one at a time and generated overlapping fragments from 13 to 25 bases long with a near neighbor ΔG energy less than -14.5. These fragments have been shown to have strong binding strength and produce unique base calls. The generated probes and sequence fragments were the input to the *in silico *model [17] for simulation which compared the fragments to the probe sets and determined the base calls a target sequence would generate. The predicted base calls were assembled into a simulated resequencing microarray result.

#### Probe sequence selection

The simulated result of a target sequence for the current prototype sequence was then run through the previously developed CIBSI analysis algorithm [8] with the following criteria. A sequence was considered detected by the current prototype sequence if at least one region of 50 or more contiguous nucleotides was predicted to consist of A, C, G, and T base calls and no ambiguous base calls (Ns). As shown in Figure 1 "Yes" for "CIBSI would identify" updated the list of sequences that could be detected by the current prototype sequence. This procedure was applied for every downloaded sequence. For example, if we collect sequences "a-z" from GenBank, we will first use sequence "a" as a prototype sequence, then use sequences "b-z" each in turn to generate target sequences for *in silico *simulation. After the completion of the simulation and CIBSI analysis, a list of sequences from the pool of sequences "b-z" that can be detected by prototype sequence "a" will be generated. Then the cycle begins again with sequence 'b" as prototype sequence, while sequences "a, c-z" each in turn is used as target sequences to generate the list for sequence "b". The cycle will continue until we generate the list for all download sequences (sequences 'a-z"). After this is completed, the second stage of the process will be undertaken. The number of sequences that a sequence (as prototype) is predicted to detect will be sorted and ordered. The sequence that was predicted to detect the most other sequences (as targets) was selected as a probe sequence to be used in the microarray design. It was then removed from the list of sequences. All the target sequences detected by that prototype sequence were also removed from the list of sequences. This procedure was repeated until the list of sequences was empty. When two or more prototype sequences were predicted to detect the same maximal number of target sequences, one was randomly selected hence the method was non-deterministic. The process was repeated with different random seeds and the number of required probe sequences did not vary significantly while the sequences used in the microarray design could change.

#### Application: HRV and HEV

The described design method could be applied to any group of sequences and a minimum set of prototype regions would be determined. The group of sequences used for HRV probe design was chosen using different criteria than those used to select the HEV sequences in the HEV probe design due to differences in the available sequences for each in GenBank. At the time of this design, only eight HRV serotypes had complete genomes sequenced. These genome sequences and all complete and partial 5'UTR sequences available for HRV in GenBank were retrieved in April 2006 and a total of 150 sequences were used in the predictive modeling. A set of 26 sequences with lengths between 145 and 500 bp were predicted to provide detection of all those input sequences (Additional file 1).

Because HEV is better characterized with complete genome sequences of all 60 recognized serotypes, the design algorithm was applied to one complete genome sequence of each serotype. In addition, the design algorithm was applied to the 3D region. The design procedure generated 4 to 8 sequences for HEV detection using 5'UTR region, and 13 sequences were predicted to detect all HEV 3D regions. It was decided to use corresponding 5'UTR sequences of the 13 genomes that the 3D targets were selected from so that the same serotype was targeted by both target regions (Additional file 1). These 13 5'UTR regions were predicted to still provide complete and now redundant coverage.

### Specificity of RPM-Flu v.30/31 chip for HRV detection and serotyping: design method 1

**Figure 2:**
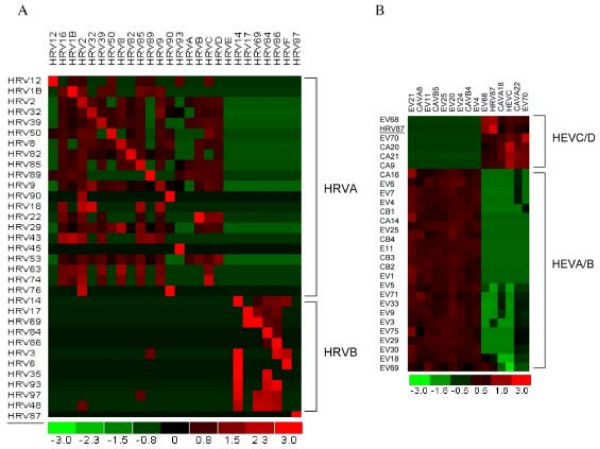
**Hybridization profiles of HRV and HEV serotypes from RPM-Flu v.30/31 microarrays.** (A) 34 HRV serotypes were classified into two clusters corresponding to species HRVA and HRVB; (B) 29 HEV serotypes (including HRV87) were classified into two clusters corresponding to HEVA/B and HEVC/D species. Base call rates (number of base calls/probe length in each tile) generated from viral samples (rows) and prototype probes (columns) were calculated and clustered using dChip software. Rows standardized base call rates. Positive hybridization was represented by red color. Higher base call rates were shown as brighter red colors. Negative hybridization (no base call) was represented by green color. The sample HRV87 is underlined.

To assess the performance of RPM-Flu v.30/31 chip design, 34 known HRV serotype strains obtained from ATCC were tested. Of the 34 strains, 18 had corresponding 5'UTR sequences tiled on the microarray and were called prototype serotypes, which were used to verify the accuracy of the designed HRV probes. The remaining 16 strains, representing near neighbor serotypes, were selected from diverse clades based on phylogenetic classification of HRV serotypes [18,19]. These strains were used to investigate the capability of the microarray to detect other HRV serotypes that did not have their sequences tiled on the microarray. Overall, the selected strains covered every single clade of 101 HRV serotypes based on phylogenetic analysis of the P1–P2 regions of 5'UTR sequences [18]. One metric of the hybridization in a reference region is to divide the number of bases reported as A, C, G, or T by the total number of bases for that region (probe length), which we refer to as the base call rate and proportionally reflects the hybridization strength or homology between the prototype and target sequences. A hybridization profile (Fig. 2A) using the base call rates clearly showed a unique pattern for each serotype. The closely related serotypes with less nucleotide divergences had similar hybridization profiles across the tiled regions, so it is possible to assign species (HRVA and HRVB) based only on the hybridization patterns. The brighter red spots (higher base call rates) along the diagonal suggested that stronger hybridizations between the tiled probes and 5'UTRs from the prototype serotypes. It is also of note that HRV87 does not fall into either of two major clusters which agrees with other findings that it should really be classified as a HEV [20,21]. To validate the accuracy of the array clustering, 5'UTR of each serotype was amplified by type-specific RT-PCR and subjected to conventional sequencing. The phylogenetic tree derived from the *de novo *5'UTR sequences (Additional file 2) confirms the HRVA and HRVB classification. Pair-wise sequence comparisons also indicated that the average nucleotide divergence of 5'UTR sequences between all 34 strains was 20.3%. The maximal nucleotide divergences among HRVA and HRVB strains were 33.4% and 32.7%, respectively. These results demonstrated that RPM-Flu v.30/31 chip is potentially capable of detecting genetically diverse HRV serotypes.

Although useful, the visual patterns are difficult to discriminate the serotypes within each species and would require generating reference patterns for all HRV serotypes. The use of sequence calls and analysis software, CIBSI [22], allows for straightforward serotype identification. The sequence analysis results showed that the identifications made by RPM-Flu v.30/31 and CIBSI are consistent with the reported typing, and could clearly differentiate most of the serotypes of the HRV (Table 1). Of the 34 HRV strains, 18 matched the selected prototype 5' UTR sequences on the RPM-Flu v.30/31 chips and showed strong hybridization signals to their corresponding prototype regions generating at least 86% base call rates. Due to the sequence homology, it is not surprising to find a noticeable and sometimes significant amount of hybridization (20 – 80%) leading to base calls on prototype regions other than the correspondent prototype for most samples. Because the identification scheme used was based on comparing the sequences generated on the array to database entries, this information could be integrated into the final identification. For these samples with high base call rates, the best hit would have tens to hundreds more matched bases than the next best hit. The remaining 16 ATCC strains represent near neighbor serotypes, which 5'UTR sequences share >80% identifies to those of prototypes. In all but the case of HRV5, the information was sufficient to identify the correct serotypes. In these samples, the difference in the number of base calls matching in the best hit and the next best hit was more variable and on average was fewer. The confidence of the identification depends on the accuracy of the base call. Since the best hit always had at least one base that matched the resequencing results and was a mismatch for the next best hit, it was possible to establish the lowest confidence level that the best hit was the correct identification. The resequencing microarray's accuracy for determining the base call has been established under a variety of conditions [23]. Using this information, there is a .000001^n ^that the next best hit is the most similar sequence in the database because a base is misidentified by the resequencing microarray where n is the number of mismatches between the next best hit and the matches for the best result. With .0001% being the largest level of uncertainty seen for these samples, it was deemed acceptable to treat the best hit as the correct identification. For HRV5, several database sequences representing different serotypes had the same score and since no further information was available it was only possible to determine that a HRV species B was present. The 5'UTR sequences from the tested strains generated with *de novo *sequencing were subjected to *in silico *predictive modeling analysis and the result of this was used as input in the CIBSI analysis program. For the case of HRV5, the *in silico *model predicted a larger fraction of base calls being made than were observed in the experiment. For all other samples there was a good correspondence on base call fractions between model and experiment as expected. This leads us to suspect there was a processing error or sample degradation leading to the less accurate identification.

**Table 1:** Identification of HRV serotypes using microarray, *de novo *sequencing, and *in silico *model of *de novo *sequence results.

Sample name	ATCC typing	Array	*de novo *sequence*	*in silico *model	Accession Number
**VR-1122**	**HRV12**	**HRV12**	**HRV12 (99%)**	**HRV12**	**EU870449**
**VR-1366**	**HRV1B**	**HRV1B**	**HRV1B (99%)**	**HRV1B**	**EU870452**
**VR-482**	**HRV2**	**HRV2**	**HRV2 (99%)**	**HRV2**	**EU870453**
**VR-1142**	**HRV32**	**HRV32**	**HRV32 (100%)**	**HRV32**	**EU870454**
**VR-340**	**HRV39**	**HRV39**	**HRV39 (99%)**	**HRV39**	**EU870455**
**VR-517**	**HRV50**	**HRV50**	**HRV50 (100%)**	**HRV50**	**EU870457**
**VR-1192**	**HRV82**	**HRV82**	**HRV82 (100%)**	**HRV82**	**EU870460**
**VR-1195**	**HRV85**	**HRV85**	**HRV85 (99%)**	**HRV85**	**EU870462**
**VR-1199**	**HRV89**	**HRV89**	**HRV89 (99%)**	**HRV89**	**EU870465**
**VR-1118**	**HRV8**	**HRV8**	**HRV8 (100%)**	**HRV8**	**EU870459**
**VR-489**	**HRV9**	**HRV9**	**HRV9 (100%)**	**HRV9**	**EU870466**
**VR-284**	**HRV14**	**HRV14**	**HRV14 (99%)**	**HRV14**	**EU870450**
**VR-1127**	**HRV17**	**HRV17**	**HRV17 (100%**	**HRV17**	**EU870451**
**VRV-1179**	**HRV69**	**HRV69**	**HRV69 (100%)**	**HRV69**	**EU870458**
**VR-1194**	**HRV84**	**HRV84**	**HRV84 (100%)**	**HRV84**	**EU870461**
**VR-1196**	**HRV86**	**HRV86**	**HRV86 (99%)**	**HRV86**	**EU870463**
**VR-1197**	**HRV87**	**HRV87**	**HRV87 (99%)**	**HRV87**	**EU870464**
**VR-1291**	**HRV90**	**HRV90**	**HRV90 (100%)**	**HRV90**	**EU870467**
VR-1128	HRV18	HRV18	HRV18 (100%)	HRV18	EU870469
VR-1132	HRV22	HRV22	HRV22 (100%)	HRV22	EU870470
VR-1139	HRV29	HRV29	HRV29 (99%)	HRV29	EU870471
VR-1153	HRV43	HRV43	HRV43 (100%)	HRV43	EU870474
VR-1184	HRV74	HRV74	HRV74 (98%)	HRV74	EU870480
VR1186	HRV76	HRV76	HRV76 (99%)	HRV76	EU870481
VR-483	HRV3	HRV3	HRV3 (99%)	HRV3	EU870472
VR-485	HRV5	HRV-B	HRV5 (100%)	HRV5	EU870476
VR-486	HRV6	HRV6	HRV6 (99%)	HRV6	EU870478
VR-508	HRV35	HRV35	HRV35 (100%)	HRV35	EU870473
VR-512	HRV45	HRV45	HRV45 (100%)	HRV45	EU870456
VR-1294	HRV93	HRV93	HRV93 (100)	HRV93	EU870468
VR-1297	HRV97	HRV97	HRV97 (93%)	HRV97	EU870482
VR-1173	HRV63	HRV63	HRV62 (90%)	HRV63	EU870479
VR-515	HRV48	HRV48	HRV48 (98%)	HRV48	EU870475
VR-1163	HRV53	HRV53	HRV53 (99%)	HRV53	EU870477

Prototype serotypes having strong hybridization signals were designated in bold characters. The strain not identified at serotype level by microarray was underlined.

*Serotype identities were made by searching 5'UTR sequences of HRV isolates against Genbank;

() indicates the highest percentage of identity to the sequence in Genbank.

### Specificity of RPM-Flu v.30/31 chip for HEV detection and serotyping: design method 2

A panel of 28 HEV serotypes, including serotypes from all four HEV species, was similarly used to validate the specificity of RPM-Flu v.30/31 for HEV detection and identification. These serotypes were originally typed based on VP1 sequences (personal communication – Steve Oberste) and the majority of them belonged to members of HEVB. The hybridization profile (Figure 2B) shows distinct clusters in a similar fashion to the HRV samples based on serotypes. In this case, HEVA and HEVB make up one cluster, while HEVC and HEVD (including HRV87) comprise a second cluster. This finding is consistent with the previously described clusters for HEV UTR sequences [24,25]. The redundancy of the targets that was a consequence of how they were selected is apparent in the more uniform response observed within each cluster to the various strains.

Analysis of sequences reported from RPM-Flu 30/31 array analysis indicated that two levels of identifications were obtained from 28 strains (Table 2). Serotype level identification was made for 11 of the 28 strains, in which 9 cases correlated with typing made by the VP1 genes. For example, HEV71 and Coxsackievirus A16 (CAVA16), known to cause hand-foot-mouth disease, were unambiguously recognized as HEV71 and CAVA16 respectively using RPM-Flu v.30/31 and analysis program. Two strains were identified by RPM-Flu v.30/31 as HEV4 and HEV5, results in agreement with the conventional sequencing of each 5'UTR. However, the strains as provided by CDC were identified as HEV6 and CAVB3 respectively, based on the VP1 region. Specific serotypes could not be identified for the remaining sixteen samples using the sequence read generated from the array. Nevertheless these samples were easily categorized into the respective species. Due to amplification problems only a subset of strains were successfully *de novo *sequenced, which showed 3 – 18% variations in 5'UTR sequences. The base call rates obtained by in *silico *predictions based on the *de novo *sequences were similar to the microarray results and the identifications agreed in all but one case.

**Table 2:** Identification of HEV serotypes using microarray, *de novo *sequencing, and *in silico *model of *de novo *sequence results.

Strain	VP1 typing^#^	Array	*de novo *sequence	*in silico *model	*Accession number*
1	CAVA16	CAVA16	ND	ND	
2	EV71	EV71	ND	ND	
3	CAVA21	CAVA21	ND	ND	
4	CAVB4	CAVB4	ND	ND	
5	EV3	EV3	EV3(95%)	CAV16	EU870485
6	EV68	EV68	EV68(99%)	EV68	EU870491
7	EV69	EV69	ND	ND	
8	EV70	EV70	ND	ND	
9	EV75	EV75	EV75(99%)	EV75	EU870493
10	EV4	EV6	EV6(93%)	EV4	EU870488
11	EV5	CAVB3	CAVB3(94%)	CAVB3	EU870489
12	EV30	HEVB	EV30(95%)	EV30	EU870486
13	CAVA14	HEVA/B	ND	ND	
14	EV1	HEVB	ND	ND	
15	EV6	HEVB	EV74(94%)	HEVB	EU870490
16	EV7	HEVB	EV30(95%)	HEVB	EU870492
17	EV11	HEVB	ND	ND	
18	EV18	HEVB	EV74(93%)	HEVB	EU870483
29	EV29	HEVB	EV80(85%)	HEVB	EU870484
20	CAVA20	HEVC	ND	ND	
21	CAVA24	HEVC	ND	ND	
22	CAVA9	HEVB	ND	ND	
23	CAVB1	HEVB	ND	ND	
24	CAVB2	HEVB	ND	ND	
25	CAVB3	HEVB	ND	ND	
26	EV25	HEVB	ND	ND	
27	EV33	HEVB	CAVA12(91%)	HEVB	EU870487
28	EV9	HEVB	ND	ND	

^#^This represents the typing provided by CDC when the samples were obtained;

*Serotype identities were made by searching 5'UTR sequences of HEV isolates against Genbank;

() indicates the highest percentage of identity to the sequence in Genbank.

## Discussion

This study demonstrated the use of an algorithm for the design of probe sets based on an *in silico *predictive model [17], developed by our group, that minimized the probes needed for detection and identification of most serotypes of HRV and HEV. The potential of using resequencing microarray for simultaneous detection and identification of highly diverse respiratory pathogens, such as HRV and HEV, was also demonstrated. The conserved nature of the 5' UTR regions of HRV and HEV genomes and the capabilities of the resequencing microarray allow serotype level identification of near-neighbor serotypes of HRV and HEV, when long (> 100 nucleotides) sequences are read from the array. Identifications can be still made for shorter length sequences to the species level particularly when the array has one or more such sequences derived from different probes.

The utility of the resequencing microarray is related to the target selection, the optimized prototype sequences represented on the array. In the case of RPM-Flu v.30/31, the selection of HRV targets has proved to be very robust. The 5'UTR has been shown in this study to be a good choice for serotyping HRV on RPM, as it performed similarly well on other platforms [13,26]. All HRV variants tested in this study could be detected and identified at least to the species level. The limited number of HRV sequences available in GenBank during the time of design of RPM-Flu v.30/31 rendered a few of the targets represented on RPM-Flu v.30/31 are shorter than 200 bp. In the past year, complete genome sequences from 46 more serotypes and another two divergent HRV'X's have been reported [6,19,27]. It will be worthwhile to update the design for the next generation of the chip.

In the case of HEV, the RPM-Flu v.30/31 assay identified only 11 of 28 strains tested at serotype level. Several strains not producing serotype identifications might have been indicative of assay protocol issues or probe design. The fact that the *in silico *model prediction was also not serotype specific indicates it was most likely a design issue. This was further confirmed by agreement in base calls made from the resequencing microarray and from conventional resequencing. Although all the strains of HEV have complete genome sequences, there are also many partial sequence submissions for each strain in GenBank that were ignored for the HEV design. A re-examination of the 5'UTR regions showed up to 14% difference in sequences grouped in the same serotype. This indicates that a redesign of the HEV prototype regions is needed where selection of a minimal set of prototype regions would be based on all available 5'UTR sequence data (complete and partial) and not a subset of genome sequences.

Comparing the identifications made from *de novo *sequencing to the identifications made by CDC (sources of the samples) illustrated another shortcoming of using the 5'UTR region for HEV that did not occur for HRV. Oberste et al. demonstrated that typing based upon HEV VP1 capsid gene sequences showed excellent correlation with serotype determined by classical antigenic methods [28]. Thus amplification and sequencing of the partial VP1 amino-terminal coding region has been accepted as a standard molecular typing method for HEV but such is not the case for the 5'UTR region [29-33]. Our results show that the 5'UTR region did not correlate as closely as VP1-based typing to antigenic type definitions for HEV unlike how it performed for HRV. While the 5'UTR region is sufficient to accurately identify the groupings, a design using VP1 as the probe region is needed to provide serotyping identifications that will match classical methods.

The current RPM design can detect and identify a more comprehensive set of viral and bacterial respiratory pathogens in parallel, including detailed discrimination of certain serotypes of HRV and HEV. This study showed that most shortcomings in the design were a result of not including adequate reference sequences for the initial design. The selection of VP1 and 3D regions also showed that incorporation of primer design considerations must be contemplated sooner in the design process than it has been currently done to prevent the selection of regions that cannot be used. Future development will address these limitations by reducing HEV probe redundancy and lack of coverage, by updating or confirming the HRV probes to be derived from newly available HRV sequences, and by involving primer design earlier in the overall design process.

## Conclusion

A powerful feature of the expanded RPM-Flu v.30/31 resequencing pathogen microarray is that the nucleotide sequences generated from hybridization of the sample RNA/DNA and array-bound probe sets in conjunction with previously developed sequence analysis algorithm CIBSI can be easily interpreted to make serotype or strain identifications. This feature and the platform's high resolution and high throughput aspects undoubtedly have great potential for use as a diagnostic tool, and therefore, efforts are currently underway to test the utility of this array on more clinical samples. The results presented also validated the usefulness of the design methodology and it is currently being applied to assist in a new microarray application associated with other genetically diverse viruses.

## Methods

### Viral strains, specimen and nucleic acid isolation

A panel of 27 cultured enterovirus (HEV) prototype strains was purchased from Center for Disease Control and Prevention (CDC, Atlanta, GA). The prototype strains of 34 rhinoviruses (HRV) and HEV69 with known titers were purchased from the American Type Culture Collection (ATCC, Manassas, VA). Total nucleic acids were extracted from 125 μl cultured samples by using the MasterPure™ DNA purification kit (Epicentre Technologies, Madison, WI) and dissolved in 20 μl of nuclease-free water.

### Primer design

All 5'UTR sequences of HRV and HEV with approximately 750 bp sizes were downloaded from GenBank. Potential PCR primer pairs that are able to amplify 600 – 700 bp fragments from HRV and HEV were automatically selected by a perl script primer search program developed by our group using the rules described in previous publication [10,11].

### Multiplex reverse transcription polymerase chain reaction

The multiplex reverse transcription polymerase chain reaction (RT-PCR) protocols for RPM-Flu v.30/31 were carried out as previously described [10] with the following modifications. For the RT step, primer LN was replaced by primer NLN (a random 9mer with the unique linker sequence), 1 pg each of two internal controls NAC1 and triosephosphate isomerase (TIM), and 5 μl of the extracted viral nucleic acids were used. The 5 μl RT reaction product was subjected to the multiplex PCR reaction. Platinum *Taq *DNA polymerase (Invitrogen Life Technologies, Carlsbad, CA) was replaced by GoTaq^® ^DNA polymerase (Promega Corporation, Madison, WI) in the PCR reaction. Primer NL instead of primer L was used with 50–150 nM each of 5'UTR primers in the multiplex PCR. The amplification reaction was carried out in a Peltier Thermal Cycler – PTC240 DNA Engine Tetrad 2 (MJ Research Inc., Reno, NV) with an initial incubation at 25°C for 10 min, then preliminary denaturation at 94°C for 2 min followed by 16 cycles of 94°C for 30 s, 45–60°C for 30 s (incremental increase of 1°C per cycle), and 72°C for 90 s, then 24 cycles of 94°C for 30 s and 60°C for 120 s.

### Microarray hybridization and analysis

Microarray hybridization and processing, and the image scanning were performed according to the manufacture's recommended protocol (Affymetrix Inc., Santa Clara, CA) using a GenChip resequencing assay kit (Affymetrix) with modification as previously described [10]. After scanning, GCOS software was used to reduce the raw image (.DAT) file to a simplified file format (.CEL file) with intensities assigned to each of the corresponding probe positions. GDAS software was then used to produce nucleotide reads (A, C, G and T) or base calls, comparing the respective intensities for the sense and antisense probe sets. The sequences from base calls made for each tiled region of the resequencing microarray were exported from GDAS as the FASTA-formatted files. Base call rate refers percentage of number of base calls generated from the full length of probe in each tile.

Final pathogen identification for the RPM-Flu v.30/31 assay was performed using Computer-Implemented Biological Sequence Identifier (CIBSI) Version 2.0 software [22], an automatic pathogen identification algorithm based on nucleic acid sequence alignment, which was developed and tested in detail in previous studies [10,11]. The NCBI BLAST and taxonomy databases used for CIBSI analysis was downloaded in December 2007. Heat-map and clustering dendrogram was made with dChip 2005 (DNA-Chip Analyzer, ). The rows of the imported data (base call rates) were standardized and clustered. Clustering distance was 1 – correlation with average linkage, and gene ordering by cluster tightness.

### DNA sequencing and analysis

5'UTR sequences were amplified from HRV- or HEV cDNA with specific primers. Amplified products were purified and sent to Macrogen USA (Gaithersburg, MD) for automated Sanger/electrophoresis-based sequencing using corresponding specific primers. Phylogenetic analysis of 5'UTR sequences was performed by using neighbor-joining method in MEGA software . All nucleotide sequences used in this study are available at GenBank (accession nos. EU870449–EU870493).

## Authors' contributions

ZW conceived and designed the study, performed microarray experiments, analyzed data and wrote the manuscript; AM designed microarray probes, analyzed data and wrote the manuscript; BL assisted in data analysis and preparing the manuscript; CK, NL and KB performed microarray experiments; DT helped to generate heatmap; CT assisted in data analyses; DS initiated the project and helped to prepare the manuscript.

## Supplementary Material

Additional file 1 **Additional Table 1.** HRV and HEV target regions on RPM-Flu v.30/31 chip. This table lists sequence information of HRV and HEV tiles on RPM-Flu v.30/31 chip. Click here for file

Additional file 2 **Additional Figure 1.** Phylogenetic analysis of the 5'UTR sequences of 31 HRV serotypes rooted with HRV87 showed the similar HRV clustering to microarray. *de novo *5'UTR sequences of 31 HRV serotypes tested in this study were subjected to phylogenetic analysis. Classification of HRVA and HRVB groups in the phylogenetic tree correlates with microarray clustering. Click here for file
