# Supplementary material for: Resequencing microarray probe design for typing genetically diverse viruses: human rhinoviruses and enteroviruses
Source: BMC Genomics. 2008 Dec 1;9:577. doi: 10.1186/1471-2164-9-577 (PMC2607299; doi:10.1186/1471-2164-9-577)
Supplement: Additional file 1 — Additional Table 1. HRV and HEV target regions on RPM-Flu v.30/31 chip. This table lists sequence information of HRV and HEV tiles on RPM-Flu v.30/31 chip. [file 1471-2164-9-577-S1.doc]

**Additional Table 1. HRV and HEV target regions on RPM-Flu v.30/31 chip**

| ALIAS | ORGANISM NAME | GENE NAME | LENGTH | ACCESSION NUMBER |
| --- | --- | --- | --- | --- |
| CAVA183D^30^ | Coxsackievirus A18 | 3D | 500 | AF499640 |
| CAVA18UTR | Coxsackievirus A18 | 5’ noncoding region | 720^30^ 700^31^ | AF499640 |
| CAVA223D^30^ | Coxsackievirus A22 | 3D | 500 | AF499643 |
| CAVA22UTR | Coxsackievirus A22 | 5’ noncoding region | 720^30^ 700^31^ | AF499643 |
| CAVA83D^30^ | Coxsackievirus A8 | 3D | 500 | AY421766 |
| CAVA8UTR | Coxsackievirus A8 | 5’ noncoding region | 720^30^ 700^31^ | AY421766 |
| CAVB43D^30^ | Coxsackievirus B4 | 3D | 500 | X05690 |
| CAVB4UTR | Coxsackievirus B4 | 5’ noncoding region | 720^30^ 700^31^ | X05690 |
| CAVB53D^30^ | Coxsackievirus B5 | 3D | 500 | AF114383 |
| CAVB5UTR | Coxsackievirus B5 | 5’ noncoding region | 720^30^ 700^31^ | AF114383 |
| EV113D^30^ | Echovirus 11 | 3D | 500 | X80059 |
| EV11UTR | Echovirus 11 | 5’ noncoding region | 720^30^ 700^31^ | X80059 |
| EV203D^30^ | Echovirus 20 | 3D | 500 | AY302546 |
| EV20UTR | Echovirus 20 | 5’ noncoding region | 720^30^ 700^31^ | AY302546 |
| EV213D^30^ | Echovirus 21 | 3D | 500 | AY302547 |
| EV21UTR | Echovirus 21 | 5’ noncoding region | 720^30^ 700^31^ | AY302547 |
| EV243D^30^ | Echovirus 24 | 3D | 500 | AY302548 |
| EV24UTR | Echovirus 24 | 5’ noncoding region | 720^30^ 700^31^ | AY302548 |
| EV253D^30^ | Echovirus 25 | 3D | 500 | AY302549 |
| EV25UTR | Echovirus 25 | 5’ noncoding region | 720^30^ 700^31^ | AY302549 |
| EV43D^30^ | Echovirus 4 | 3D | 500 | AY302557 |
| EV4UTR | Echovirus 4 | 5’ noncoding region | 720^30^ 700^31^ | AY302557 |
| EVCUTR | Enterovirus C | 5’ noncoding region | 331 | AF542451 |
| EV683D^30^ | Echovirus 68 | 3D | 500 | AY426531 |
| EV68UTR | Echovirus 68 | 5’ noncoding region | 720^30^ 700^31^ | AY426531 |
| EV703D^30^ | Echovirus 70 | 3D | 500 | NC_001430 |
| EV70UTR | Echovirus 70 | 5’ noncoding region | 700 | NC_001430 |
| HRV12UTR | Human rhinovirus | 5’ noncoding region | 146 | AY371986 |
| HRV14UTR | Human rhinovirus | 5’ noncoding region (and VP1)^30^ | 1200^30^ 500^31^ | K02121 |
| HRV16UTR | Human rhinovirus | 5’ noncoding region (and VP1)^30^ | 1200^30^ 500^31^ | L24917 |
| HRV17UTR | Human rhinovirus | 5’ noncoding region | 147 | AY371988 |
| HRV1BUTR | Human rhinovirus | 5’ noncoding region (and VP1)^30^ | 1200^30^ 500^31^ | D00239 |
| HRV2UTR | Human rhinovirus | 5’ noncoding region (and VP1)^30^ | 1200^30^ 500^31^ | X02316 |
| HRV32UTR | Human rhinovirus | 5’ noncoding region | 144 | AY371975 |
| HRV39UTR | Human rhinovirus | 5’ noncoding region (and VP1)^30^ | 1200^30^ 500^31^ | AY751783 |
| HRV50UTR | Human rhinovirus | 5’ noncoding region | 145 | AY371978 |
| HRV69UTR | Human rhinovirus | 5’ noncoding region | 316 | AF542426 |
| HRV82UTR | Human rhinovirus | 5’ noncoding region | 177 | AY371976 |
| HRV84UTR | Human rhinovirus | 5’ noncoding region | 324 | AF542429 |
| HRV85UTR | Human rhinovirus | 5’ noncoding region (and VP1)^30^ | 1200^30^ 500^31^ | HRV-85* |
| HRV86UTR | Human rhinovirus | 5’ noncoding region | 315 | AF542431 |
| HRV87UTR | Human rhinovirus | 5’ noncoding region | 572 | AY062273 |
| HRV89UTR | Human rhinovirus | 5’ noncoding region (and VP1)^30^ | 1200^30^ 500^31^ | A10937 |
| HRV8UTR | Human rhinovirus | 5’ noncoding region | 145 | AY371983 |
| HRV90UTR | Human rhinovirus | 5’ noncoding region | 147 | AY371974 |
| HRV93UTR | Human rhinovirus | 5’ noncoding region | 474 | AF108152 |
| HRV9UTR | Human rhinovirus | 5’ noncoding region (and VP1)^30^ | 1200^30^ 500^31^ | HRV-9* |
| *HRVAUTR | Human rhinovirus | 5’ noncoding region | 147 | AY371943 |
| HRVBUTR | Human rhinovirus | 5’ noncoding region | 149 | AY371949 |
| HRVCUTR | Human rhinovirus | 5’ noncoding region | 144 | AY371931 |
| HRVDUTR | Human rhinovirus | 5’ noncoding region | 147 | AY371964 |
| HRVEUTR | Human rhinovirus | 5’ noncoding region | 149 | AY371972 |
| HRVFUTR | Human rhinovirus | 5’ noncoding region | 146 | AY371937 |

**Note:** Superscripts indicate on which version of the chip the sequences are present.

*****HRV9 and HRV85 sequences were downloaded from [http://www.picornaviridae.com](http://www.picornaviridae.com/).
