# Supplementary material for: Resequencing microarray probe design for typing genetically diverse viruses: human rhinoviruses and enteroviruses
Source: BMC Genomics. 2008 Dec 1;9:577. doi: 10.1186/1471-2164-9-577 (PMC2607299; doi:10.1186/1471-2164-9-577)

Additional Figure 1. Phylogenetic analysis of the 5’UTR sequences of 31 HRV serotypes rooted with HRV87 showed the similar HRV clustering to microarray.


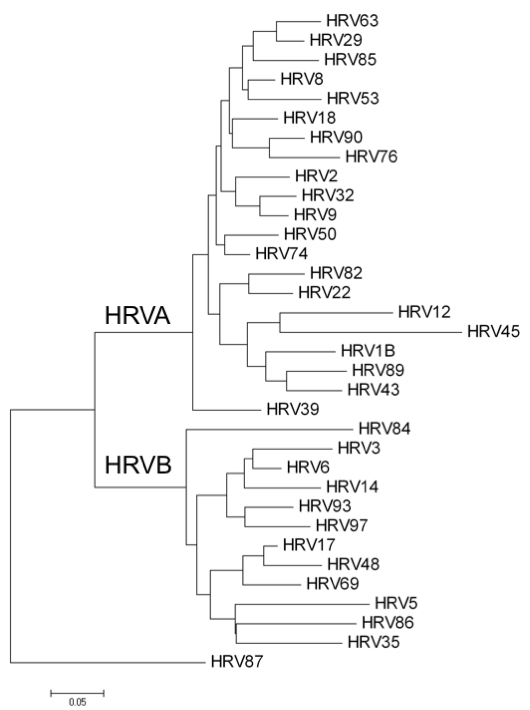

Supplement: Additional file 2 — Additional Figure 1. Phylogenetic analysis of the 5'UTR sequences of 31 HRV serotypes rooted with HRV87 showed the similar HRV clustering to microarray. de novo 5'UTR sequences of 31 HRV serotypes tested in this study were subjected to phylogenetic analysis. Classification of HRVA and HRVB groups in the phylogenetic tree correlates with microarray clustering. [file 1471-2164-9-577-S2.doc]
